# Antibodies to the α_1_-Adrenergic Receptor Cause Vascular Impairments in Rat Brain as Demonstrated by Magnetic Resonance Angiography

**DOI:** 10.1371/journal.pone.0041602

**Published:** 2012-07-30

**Authors:** Peter Karczewski, Andreas Pohlmann, Babette Wagenhaus, Natali Wisbrun, Petra Hempel, Bernd Lemke, Rudolf Kunze, Thoralf Niendorf, Marion Bimmler

**Affiliations:** 1 E.R.D.E.-AAK-Diagnostik GmbH, Berlin, Germany; 2 Berlin Ultrahigh Field Facility (B.U.F.F.), Max Delbrueck Center for Molecular Medicine, Berlin, Germany; 3 Animal Facilities, Max Delbrueck Center for Molecular Medicine, Berlin, Germany; 4 IT Department, Max Delbrueck Center for Molecular Medicine, Berlin, Germany; 5 E.R.D.E. e.V., Berlin, Germany; 6 Experimental and Clinical Research Center, a joint cooperation between the Charité Medical Faculty and the Max Delbrück Center for Molecular Medicine, Berlin, Germany; 7 Autoimmunity and G Protein-Coupled Receptors, Max Delbrueck Center for Molecular Medicine, Berlin, Germany; University of Manchester, United Kingdom

## Abstract

**Background:**

Circulating agonistic autoantibodies acting at G protein-coupled receptors have been associated with numerous sever pathologies in humans. Antibodies directed predominantly against the α_1_-adrenergig receptor were detected in patients suffering from widespread diseases such as hypertension and type 2 diabetes. Their deleterious action has been demonstrated for peripheral organs. We postulate that antibodies to the α_1_-adrenergig receptor are relevant pathomolecules in diseases of the central nervous system associated with vascular impairments.

**Methodology/Principal Findings:**

Using a rat model we studied the long-term action of antibodies against the α_1_-adrenergig receptor either induced by immunization with a receptor peptide or applied by intravenous injection. The vasculature in the rat brains was investigated by time-of-flight magnetic resonance angiography using a 9.4 Tesla small animal MR imaging system. Visual examination of maximum-intensity-projections (MIPs) of brain angiographs revealed the development of vascular defects in antibody- exposed animals between three and eight months of treatment. Relative vascular areas were derived from representative MIP image sections by grayscale analysis and used to form an index of vascular circulation. Animals exposed to the action of α_1_-adrenergig receptor antibodies showed significantly reduced vascular areas (p<0.05). Calculated index values indicated attenuated blood flow in both antibody-treated cohorts compared to their respective controls reaching with (relative units ± standard error, n = 10) 0.839±0.026 versus 0.919±0.026 statistical significance (p<0.05) for peptide-immunized rats.

**Conclusion/Significance:**

We present evidence that antibodies to the α_1_-adrenergig receptor cause cerebrovascular impairments in the rat. Our findings suggest the pathological significance of these antibodies in pathologies of the human central nervous system linked to impairments of brain vasculature such as stroke and dementia.

## Introduction

Structural and functional impairments of the vasculature are causally linked or significantly contribute to different pathologies in humans, among them numerous widespread diseases. In the cardiovascular system hypertension, angina pectoris and cardiac infarction involve vascular impairments. Patients suffering from diabetes develop vascular injuries. Severe defects of blood supply to and circulation in the brain are the acute cause of stroke. Brain vasculature is critical for the development of different types of dementia such as Alzheimer’s and vascular dementia. There is evidence that dementia of the Alzheimer’s type may be primary a vascular disease [1]. Thus, damages in the blood vessel system represent a significant factor in the development and progression of numerous severe diseases.

Agonistic autoantibodies acting at G protein-coupled receptors (GPCR) have been detected in the circulation of patients with different, mainly cardiovascular diseases [2], [3]. These antibodies bind to epitopes localized at the extracellular loops of GPCR, thereby activating the receptor system in a similar but not identical manner as the physiological agonists. They may disable protective mechanisms of the target cell such as receptor desensitization resulting in prolonged, unphysiological activation of receptor pathways [4]. Their pathogenic potential was demonstrated in animal models and in clinical studies [5]–[9]. Agonistic autoantibodies to the α_1_-adrenergic receptor (α_1_-AR) were found to be associated with widespread diseases such as different types of hypertension and type 2 diabetes [9], [10]–[12]. Antibodies to the α_1_-AR were shown to cause cardiomyocyte hypertrophy and diastolic dysfunction in rats [7], [13]. In patients with refractory hypertension the removal of antibodies to α_1_-AR by immunoadsorption resulted in a significant and long-lasting decline of the mean arterial blood pressure [9]. Considering the central role of α_1_-AR in the regulation of blood vessels, the occurrence of antibodies acting at this receptor in diseases with significant vascular involvements suggests their importance in vascular pathology [14]. Rats immunized with α_1_-AR peptides developed receptor-specific antibodies and damages in the aorta and mesenteric artery [8].

The present investigation aimed at shedding light on the potential of α_1_-AR antibodies to cause damages in the vasculature of the central nervous system. We therefore studied the long-term effects of α_1_-AR antibodies in vital rats by time-of-flight magnetic resonance angiography (TOF-MRA) using a 9.4 Tesla small animal magnetic resonance imaging system. We observed substantial attenuations of vascular blood flow in the brain after long-term exposure to the α_1_-AR antibody.

## Materials and Methods

### Ethics Statement

Animal experiments were carried out in accordance with the guidelines provided and approved by the animal welfare department of the *Landesamt für Gesundheit und Soziales Berlin* (Berlin State Office of Health and Social Affairs, Permit Number: G0197/10). Taking blood samples and imaging experiments were performed under isoflurane anesthesia. All manipulations of animals were performed by authorized personnel, and all efforts were made to minimize suffering of animals.

### Animals and Housing Conditions

Forty male Wistar rats (10–13 weeks of age, 280–350 g) were obtained from Charles River Laboratories, Sulzfeld, Germany. Animals were housed in one acclimatized windowless indoor room in standard IVC cages Type 1500 (Techni-Plast, Sulingen, Germany) with a wire mesh top in groups of maximum 3 animals dependent on weight. The animals had free access to commercial rodent food, and tap water was available ad libitum. The environmental conditions were held constant, room temperature 20+2°C and 50–60% relative humidity. The animals were allowed to acclimatize before studies took place.

### Materials

The synthetic peptide PAPEDETICQINEE (BioSyntan, Berlin, Germany) corresponding to the second extracellular loop of the human α_1_-AR isoform A was coupled to bovine serum albumin (BSA) by the glutaraldehyde method. The peptide-BSA reaction product was desalted and pre-buffered to phosphate buffered saline (PBS) using Sephadex-G25 columns (PD-10 columns, GE Healthcare, UK). For controls, BSA fractions were prepared the same way except the peptide was absent. Antiserum to the α_1_-AR peptide was produced in goat (BioGenes, Berlin, Germany). The antiserum was affinity purified by column chromatography using the peptide immobilized to an agarose matrix (α_1_-AR antibody). Goat control immune gamma globulin was obtained from Dianova, Hamburg, Germany (control IgG).

### Immunization and Antibody Analysis

To four experimental cohorts 10 rats each were allocated at random. One cohort (AB) obtained intravenous injections of α_1_-AR antibody (700 µg/kg body weigh). The corresponding control group (C-AB) was injected with the same dose of control IgG. The injections were repeated monthly. A third cohort of animals (PEP) was immunized by subcutaneous injection of 300 µg α_1_-AR-peptide coupled to BSA and emulsified in incomplete Freund’s adjuvance at 0, 2 and 4 weeks. Then the injections were repeated monthly. The respective control animals (C-PEP) were subcutaneously injected with BSA. Blood aliquots were taken from anesthetized animals by retro-orbital sampling. The obtained sera were analyzed for the presence of α_1_-AR antibodies by standard ELISA techniques. For detecting the systemic immune response and the applied goat α_1_-AR antibody (cohorts AB and C-AB), aliquots of rat serum were bound to 96-well plates (Perbio Science, Bonn, Germany) and probed with anti-rat IgG and anti-goat IgG, respectively. To specifically detect α_1_-AR antibodies generated in immunized rats (cohorts PEP and C-PEP), the immobilized α_1_-AR peptide was used as capture antigen. As secondary antibody horseradish peroxidase conjugated anti-rat IgG or anti-goat IgG was used as appropriate. Antibody binding was visualized by the 1-Step Ultra TMB ELISA (Perbio Science, Bonn, Germany). The absorbance was measured at 450 nm with a SLT Spectra multiplate reader (TECAN, Crailsheim, Germany).

### Time-of-flight Magnetic Resonance Angiography

For the imaging experiments animals were anesthetized in a warmed anesthetic chamber using 3.5% isoflurane in an oxygen/air mixture (300∶700) with a flow rate of 1000 ml/min. After approximately one minute the isoflurane was reduced to 2% to 3%. A body temperature of 37°C was maintained throughout the experiments by warming the animal bed using a circulating heated water system (Thermo Haake GmbH, Karlsruhe, Germany) and monitoring by a rectal temperature probe. Respiratory signals were continuously monitored using a commercial monitoring and gating system (SA Instruments, Inc., New York, USA) and kept between 50–70 respiration cycles per minute by regulating the isoflurane dose.

All imaging experiments were carried out on a 9.4T small animal MR system (Biospec 94/20, Bruker Biospin, Ettlingen, Germany). A curved four channel receive only rat brain coil array (Bruker Biospin, Ettlingen, Germany) was used in conjunction with a linear polarized birdcage resonator for transmission (Bruker Biospin, Ettlingen, Germany; inner diameter of 72 mm). Due to the large increase in body size with age a shorter head-only birdcage resonator (in-house built; inner diameter of 75 mm) was used for transmission at 11 months.

For anatomical reference and to screen for possible pathologies (morphological, hemorrhage, edema) a high resolution T_2_-weighted RARE sequence (TE/TR = 33 ms/2723 ms, field of view = 35 mm, matrix size = 384×384, voxel size = 91×91×500 µm^3^, 8 averages) was acquired in axial orientation. Time-of-flight angiograms were acquired using a 2D FLASH sequence (TE/TR/FA = 3.9 ms/18 ms/90°, matrix size = 384×384, 198 image slices, field of view = 38.4×38.4, voxel size = 100×100×150 µm^3^) in coronal orientation.

### Image Processing

To analyze TOF-MRA measurements maximum-intensity-projections (MIPs) were produced using the OSIRIS software (version 4.07, Digital Imaging Unit, University Hospital of Geneva, Schweiz). In the two-dimensional viewer mode images were virtually screened using coronal MIP-slabs of three millimeter thickness to find the appropriate image section for visual evaluation. This interactive approach aimed at defining the corresponding section plane of TOF-MRA measurements of each animal at 3 months and 11 month for comparison. All efforts were made to select the same brain section and angle of view for the two-dimensional MIPs at both time points for each animal. The respective two-dimensional MIP image, which was subsequently used for grayscale analysis, was checked against the three-dimensional MIP view of the entire volume covered by the TOF-MRA to verify the visually observed alterations and to exclude artifacts due to spatial variations in brain vascular architecture.

### Data Processing/grayscale Analysis

Images were analyzed using the ImageJ software (version 1.46, Wayne Rasband, National Institute of Health, USA). Edge-preserving median filtering was performed for smoothing and to remove background noise. For each two-dimensional image section basic statistical parameters were calculated. The vascular fractions of images were differentiated based on the assumption that the most frequent and highest ranked median gray value represents the image background consisting of tissue not perfused with blood. Grey values above median plus two standard deviations were considered to correspond to the vascular fraction of the image. A threshold operation was performed to obtain black and white images. White areas representing the vascular fractions were estimated using the histogram. Changes in brain vascular circulation are expressed as ratio of the relative vascular areas of the last and the first TOF-MRA.

### Statistics

Data were analyzed using GraphPad Prism version 3.00 for Windows (GraphPad Software, San Diego California USA). Statistical significances were calculated with the unpaired Student’s t-test after data had been checked for normal distribution. Differences with p-values<0.05 were considered statistically significant.

## Results

### Induction of Antibodies to the α_1_-adrenergic Receptor in Rats

**Figure 1:**
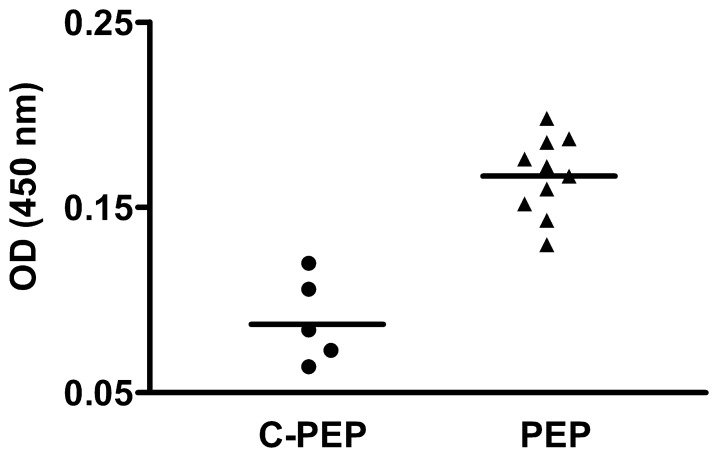
Analysis of rat sera for the presence of antibodies to the α_1_-AR. Antibodies were detected by ELISA. (PEP) rats immunized with the α_1_-AR peptide, (C-PEP) controls treated with vehicle. Blood samples were taken about one month before the final TOF-MRA. The obtained sera were analyzed at a 1∶50 dilution.

Animals receiving intravenously α_1_-AR-antibodies produced in goat (cohort AB) showed a fast clearance of these antibodies from the circulation. Six hours after injection of α_1_-AR-antibodies there was still a high level of the antibody detectable. Then the α_1_-AR-antibody level declined to less than 10% of the 6-hour value at day 1 and was not detectable in most animals after day 3. The peptide used for immunizing rats (cohort PEP) corresponds to the cell surface epitope of the rat α1-AR which is permanently exposed to the immune system. To ensure that there is an immune reaction to the application of the autoantigen, the IgG status of the animals was measured. Compared to the pre-immune situation, there was an increase in IgG. Thus, the immunization procedure was efficient and resulted in a systemic immune response. Analysis of the specific immune response to the peptide antigen demonstrated low levels of α_1_-AR-specific antibodies in serum samples of the animals. **Figure 1** illustrates the situation of α_1_-AR antibody formation in peptide- (PEP) and vehicle-immunized rats (C-PEP) one month before the final TOF-MRA measurement.

### Time-of-flight Angiography (TOF-MRA) of Rats

The total period of observation was 11 months. During this time span there was a massive growth of the rats resulting in more than doubling of their body mass. The increase within the first three months of the observation was with 60% much higher than between month three and month 11 with 26%. Considering previous experiences and data from comparable animal studies of other groups, vascular alterations were unlikely to be visible already after three months of treatment [5]–[8]. Therefore TOF-MRA images taken at month 3 and at month 11 after the beginning of the treatment were used for evaluation. Thereby we thought to reduce the impact of developmental changes for size and architecture of brain vasculature and thus to improve comparability of angiographs taken at different time.

**Figure 2:**
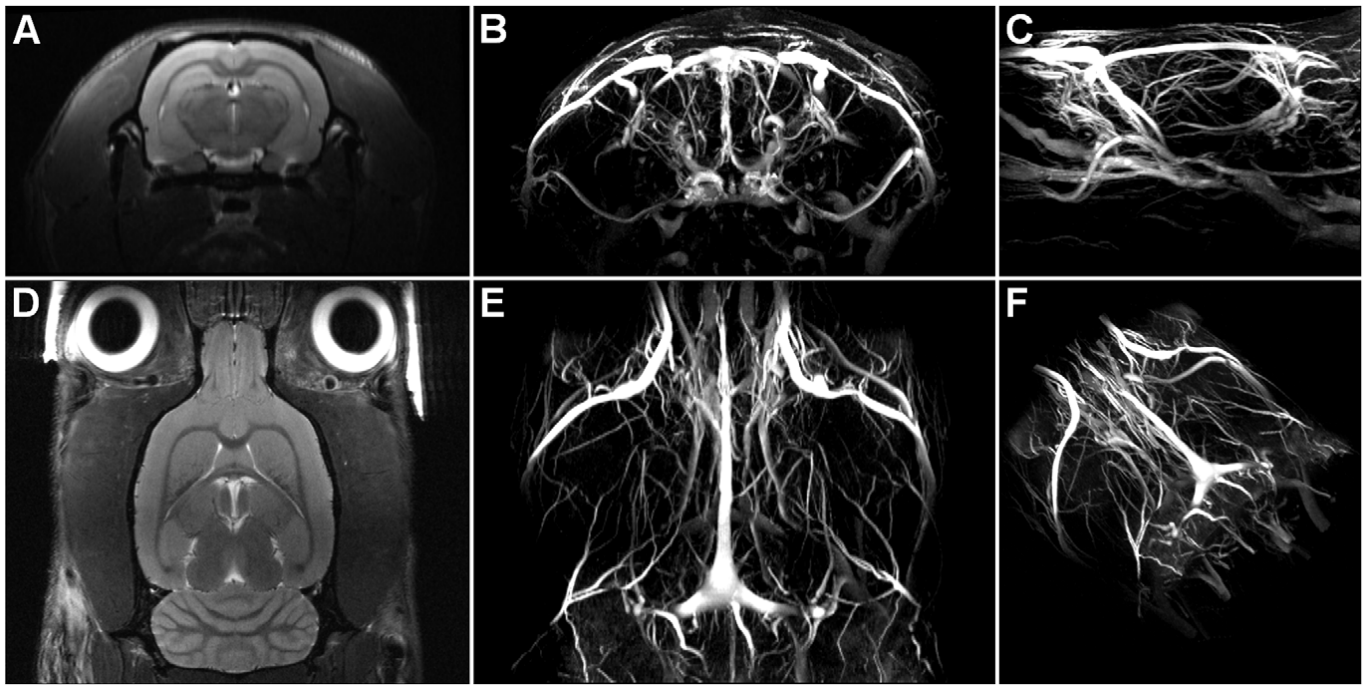
Anatomical images and TOF-MRA of the rat brain. Maximum-intensity-projections (MIP) of the TOF-MRA image data are shown for a coronal view (B) and axial view (E) together with corresponding 2-dimensional anatomical T_2_-weighted image slices (A, D). C) sagittal MIP view. F) double-oblique MIP view from the left posterior perspective (shown at a smaller scale).

Anatomical magnetic resonance images of the rat brain and maximum-intensity-projections (MIP) of corresponding TOF-MRA data are shown in **Figure 2**. The vasculature inside and outside of the brain was very well depicted.

#### Visual image evaluation

**Figure 3:**
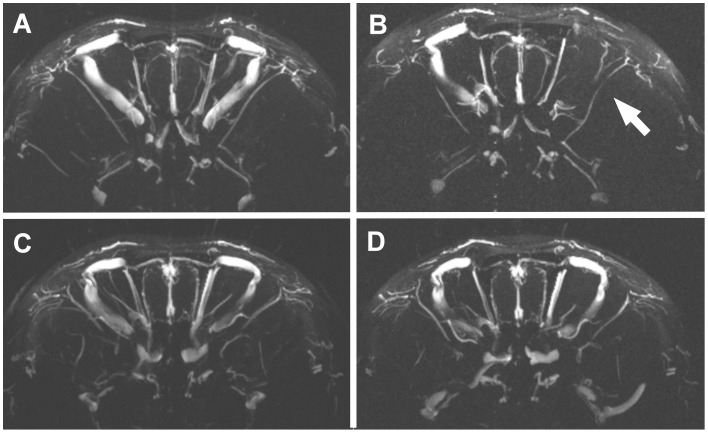
Two-dimensional maximum-intensity-projections of **TOF-MRA of rat brain.** Coronal images of rat brains were screened in 3 mm slices. The rat shown in A, B was immunized with the α_1_-AR peptide, the rat shown in C, D obtained vehicle as control. Injections were performed monthly. Angiographs of the same animal were taken at the beginning of the treatment (A, C) and eight months later (B, D). Light contrasts correlate with vascular blood flow. Eight months of treatment with the α_1_-AR peptide led to apparent attenuations of blood flow (B, right part of brain section) compared to the initial situation (A). Control injections did not result in comparable defects in the same time frame (D versus C).

Visual screening of TOF-MRA MIP images revealed impairments of vascular blood flow in the anterior part of the brain in most of the animals of both cohorts exposed to α_1_-AR antibody action within eight months of observation. In the cohort of rats immunized with the α_1_-AR peptide (PEP) 8 out of 10 animals showed visible vascular defects which were abundantly clear in four individuals. Notably, larger vessels in the right part of the brain appeared to be preferentially affected. **Figure 3** **A, B** exemplifies coronal two-dimensional MIP sections approximately at the level of the hypothalamus taken at the early stage of immunization with the α_1_-AR -peptide (**A**) and at the end of the treatment eight months later (**B**). When comparing the images regional damages in blood flow were obvious which developed within eight months of repeated immunizations with the α_1_-AR -peptide (**B** versus **A**). **Figure 3** **C, D** shows an example of comparable two-dimensional views of a brain from a control animal. In this case, no defects were detectable at the end of the observation period. However, 3 out of 10 animals in the control group (C-PEP) also showed brain regions with impaired blood flow. Although they appeared less pronounced when judged visually, quantifiable measures are required to verify these observations.

**Figure 4:**
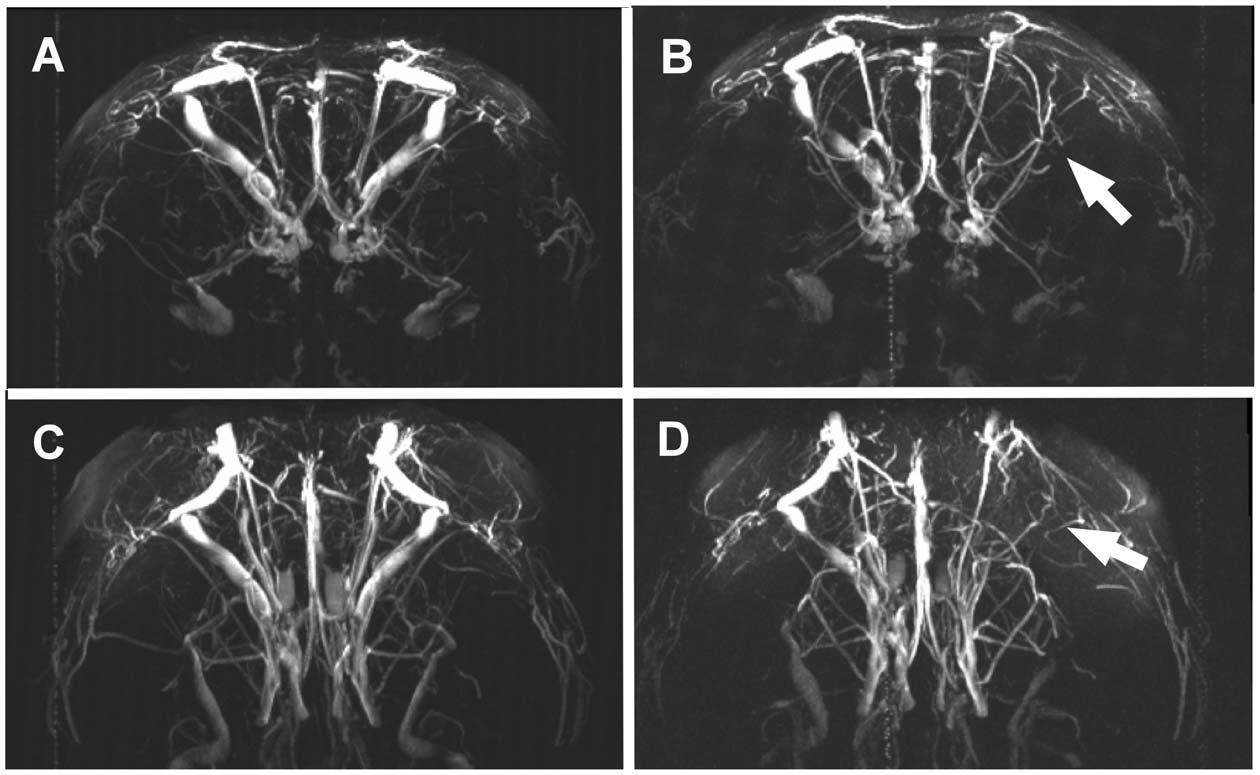
TOF-MRA of rat brain. Three-dimensional coronal views of the frontal brain segment. The rat shown is as in Figure 3 which was immunized with the α_1_-AR peptide. Angiographs of the same animal were taken at the beginning of the treatment (A, C) and eight months later (B, C). C, D show the same brain segment as in A, C but vertically rotated by 45°. The defects in blood flow in the right part of the brain as displayed in Figure 3 are obvious (B, D) compared to the situation eight months before (A, C).

Visual evaluation of TOF-MRA MIP sections of rats injected with α_1_-AR antibodies (cohort AB) showed a similar situation as obtained for the α_1_-AR peptide-treated animals. Comparing the two-dimensional MIP sections of the same individual, there were clearly vascular defects visible at 11 months after the beginning of the treatment in 9 out of 10 animals with 4 rats showing only slightly affected blood flow. As in peptide-treated rats, the impairment of different, predominantly larger brain vessels was obvious. In the respective control group (C-AB) which received control IgG, 3 out of 9 animals developed clearly visible defects. Three-dimensional MIPs of brain TOF-MRA as depicted in **Figure 4** for the peptide-treated animal were compared with two-dimensional MIP sections. The three dimensional display confirmed the observed defects and ruled out errors due to possible spatial variations of the brain vascular tree.

#### Quantitation of vessel areas by grayscale analysis

**Figure 5:**
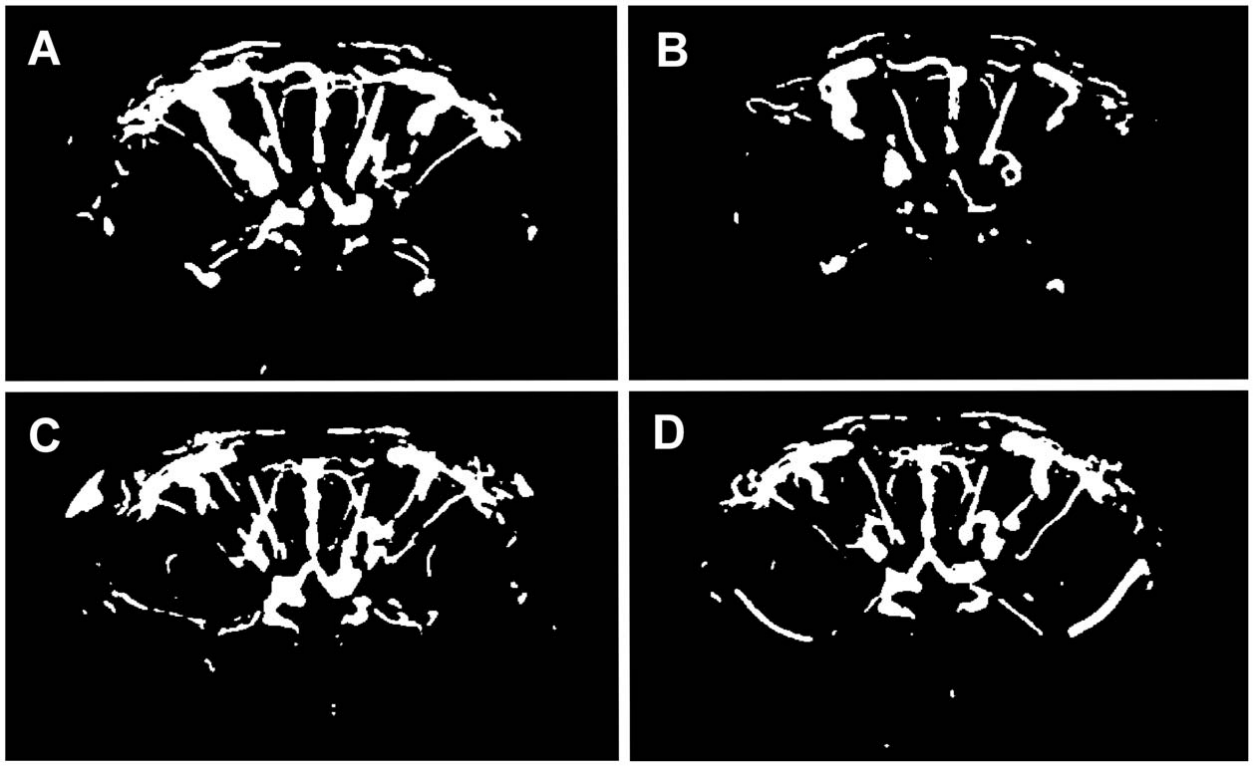
Black and white threshold images. Two-dimensional sections of maximum-intensity-projections (MIP) of TOF-MRA images were processed as described in Materials and Methods. The black and white images were generated using the median plus two standard deviations as threshold value. White areas correspond approximately to the area of vessels with blood flow. Exemplarily shown is one animal treated with intravenous injections of the α_1_-AR antibody (A, B) and one control animal (C, D) at measurement 1 (A, C) and at measurement 2 eight months later (B, D). The reduction of the white areas in B compared to A is evident.

**Figure 6:**
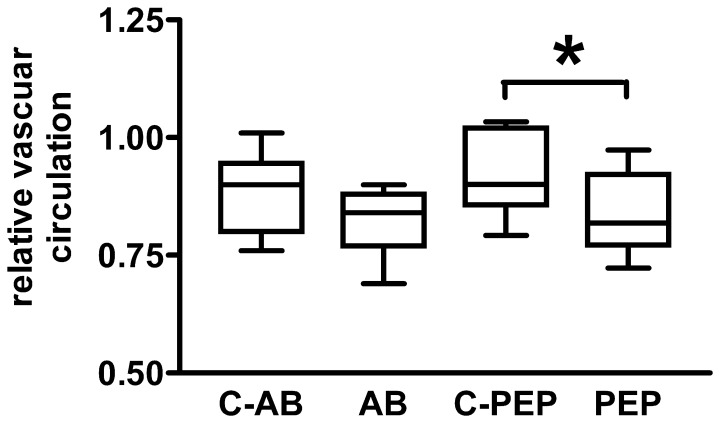
Box plot of relative brain vascular blood flow. Vascular area was calculated from two-dimensional section of angiographs of rat brains by grayscale analysis as described in the methods section. Relative vascular circulation of the individual animal is expressed as ratios of vascular areas from the last and the first TOF-MRA. Time between measurements was eight months. Ratios <1 indicate attenuated vascular blood flow. Data are given of animals treated with intravenous injections of the antibody to the α_1_-AR (AB; n = 9), their controls (C-AB); n = 10), and rats injected subcutaneously with the α_1_-AR peptide (PEP; n = 10) and the respective controls (C-PEP; n = 10). Asterisk indicates statistically significant differences with p<0.05.

Gray scale analysis of two-dimensional TOF-MRA MIP sections was performed to approximate relative values of vascular areas. An essential step of the employed procedure is the generation of black and white images. An example is given in **Figure 5** for an animal injected with the α_1_-AR antibody (**Figure 5** **A, B**) and a control animal (**Figure 5** **C, D**). The white areas correspond to blood perfused vessels. Their quantification produces a relative measure for the area of the functionally intact vasculature. **Table 1** gives the calculated mean relative vascular areas for the experimental groups. Comparing the values obtained from measurement 1 and measurement 2 eight months later, there was a small, but statistically significant reduction of vessel area within the observation period for both, rats treated with the α_1_-AR antibody (AB) and rats immunized with the α_1_-AR peptide (PEP). The respective control animals (C-AB, C-PEP) showed slightly, but not significantly decreased mean vascular areas at the final measurement. To compensate for differences between the experimental groups already obvious at the beginning of the observation, the ratio of relative vascular areas of the final measurement 2 and of the initial measurement 1 was calculated for each animal. This ratio served as an index for changes of vascular blood flow and was used to compare the animal cohorts exposed to α_1_-AR antibody action (AB, PEP) with their respective control cohorts (C-AB, C-PEP). Values <1 indicate impaired blood flow. The box plot in **Figure 6** depicts these data of brain relative vascular circulation. Both treatment groups, rats injected with the α_1_-AR antibody (AB) and rats immunized with the α_1_-AR peptide (PEP) showed larger decrements compared to the respective control animals (C-AB, C-PEP). The decline in the α_1_-AR peptide- treated cohort (PEP) was with (relative units ± standard error) 0.839±0.026 versus 0.919±0.026 of the C-PEP control cohort statistically significant (p<0.05). There was a clear tendency towards attenuated brain blood circulation also in the α_1_-AR antibody- treated animals which, however, did not reach statistical significance (0.822±0.021 for AB versus 0.877±0.029 for C-AB). Both control cohorts showed values of relative vascular circulation slightly below 1 indicating some worsening of blood supply in the brain during the observation period.

**Table 1:** Comparison of relative vascular areas.

Experimental group	Relative vascular area	
	Measurement 1	Measurement 2
C-AB (9)	9.96±0.55	8.67±0.43
AB (10)	10.49±0.36	8.65±0.42*
C-PEP (10)	9.97±0.26	9.17±0.34
PEP (10)	10.20±0.35	8.59±0.47*

Cohorts of rats were treated either with intravenous injections of α_1_-adrenoceptor antibody (AB) or with subcutaneous injections with α_1_-adrenoceptor peptide (PEP). Control animals received unspecific control IgG (C-AB) or albumin (C-PEP). Applications of substances were repeated monthly. The time span between TOF-MRA measurement 1 and measurement 2 was eight months. Relative vascular areas were determined by grayscale analysis of two-dimensional TOF-MRA maximum-intensity-projections. Values are given as means ± standard error of the mean. The number of animals of each group is given in brackets.

* significantly different to measurement 1 (p<0.05).

## Discussion

We demonstrate first evidence of the pathogenic significance of antibodies to the α_1_-AR in the brain vasculature of rats by TOF-MRA. Two parallel approaches, immunization of animals with the α_1_-AR peptide and injection of the α_1_-AR antibody, resulted in impairments of brain blood flow.

For immunizing animals we used a peptide exactly corresponding to the extracellular region of the α_1_-AR which contains the antibody epitope. This approach differs from previous studies with regard to the peptides used for immunization. In these studies longer α_1_-AR peptides were used containing amino acid stretches of transmembrane regions of the receptor [7], [8], [13]. The extracellular receptor region, the peptide’s amino acid sequence was derived from, is exposed to and normally tolerated by the immune system of the animal. Therefore the peptide which represents an autoantigen may be critical to induce antibody production when used for immunization. This may explain the relatively low α_1_-AR antibody levels of the animals observed in the present study although the generalized immune response demonstrated the efficiency of the immunization procedure. In a parallel approach, rats were treated by intravenous injection of an antibody raised against the α_1_-AR peptide. This antibody we previously characterized to be agonistic and to act comparable to α_1_-AR antibodies from hypertensive patients [9], [15]. The intravenously injected α_1_-AR antibody raised in goat disappeared fast from the circulation of the rats. Human IgG applied to mice was reported to persist in the circulation and was found with 25% recovery at four days after injection [16]. For an antiserum to a β_1_-adrenergic receptor domain raised in rats and then transferred into naive rats, a half-life time in the circulation of nine days was reported [5]. In the present study the α_1_-AR antibody applied to the rats was generated in a different animal species, and a fast clearance of the antibody by the immune system must be expected. However, the serum concentration of the antibody does not necessarily reflect the situation at its binding sites. Low levels of the antibody were detectable up to two days in the circulation of the animals and were obviously sufficient to act at the α_1_-AR of the target tissue. Data on retention time at the receptor and stability of the receptor bound antibody in vivo are not available yet.

TOF-MRA allows the depiction of the vascular tree by producing images, in which flowing blood appears bright, while the surrounding stationary tissue is suppressed and appears dark. The amount of flow-related enhancement depends on several factors, including tissue specific magnetic resonance (MR) parameters like the spin-lattice relaxation time T_1_, measurement protocol specific MR parameters (flip angle, repetition time), and geometrical parameters like image slice thickness and image orientation, or blood flow velocity. Assuming the T_1_ of blood and all MR acquisition parameters being the same in all experiments, the enhancement of blood is primarily flow-related and allows for the assessment of vascular impairments, such as reduced flow velocity or turbulences.

The most striking defects visible in TOF-MRA MIP sections from animals exposed to the action of α_1_-AR antibodies concerned larger vessels. Endothelial cells and smooth muscle cells express α_1_-AR and thus present binding sites for agonistic autoantibodies in small and large vessels. The unphysiological stimulation of α_1_-AR pathways by agonistic autoantibodies results in pathological changes of cell functions and eventually may lead to substantial cell death and thus to severe damages of vessel structure. Agonistic autoantibodies to the α_1_-AR were demonstrated to induce hyperplasia of smooth muscle in large vessels such as aorta and mesenteric arteries accompanied by the reduction of the vessel lumen [8]. Furthermore, impairments of the vascular endothelial may generate foci for clot formation. The resulting damages are likely to cause the massive attenuation or even cessation of blood flow as depicted by TOF-MRA also in large vessels. Signal intensity in TOF-MRA depends on the velocity of flowing blood. Zero blood flow gives no signal and thus results in the apparent disappearance of the afflicted vessel as shown in Figures 3 and 4. However, more information on the morphological state of the vessel can not be obtained by TOF-MRA.

To affirm the visually detected defects in brain vessels of animals exposed to the action of the α_1_-AR antibodies, relative correlates of vascular circulation were obtained by grayscale analysis of TOF-MRA MIPs. A crucial and critical step to differentiate vessels from surrounding brain tissue was the threshold operation using the median plus two standard deviations. This operation robustly eliminated noise and tissue background, but implied the loss of some smaller or less perfused vessels. The rat is known for the large potential to develop collateral vessels [17]. This ability contributes to the high degree of variability in the architecture of rat brain vasculature. The elimination of some smaller vessels, which likely comprise also newly developed collaterals, may improve the comparability of the brain vascular tree of the same animal at different ages. The reduction of relative vascular areas between the first and the final TOF-MRA within the individual experimental groups was significant for both cohorts of animals exposed to the action of α_1_-AR antibody. Index values indicated significant attenuation of vascular circulation in α_1_-AR peptide-immunized rats compared to controls. Considering the loss of a fraction of smaller vessels due to the stringent threshold operation, the observed deleterious effects on brain vasculature may be still underestimated. Furthermore, hemodynamic changes as result of the reduction of vessel lumen, which was reported to be part of α_1_-AR antibody action, are not addressed by the techniques employed in the present study [7].

Data also clearly demonstrate the tendency for impaired circulation in the brain of both control cohorts at the end of the treatment period. This is in line with the visual observations of vascular defects in some of control animals. Worsening of blood supply in the brain may be attributed to degenerative processes known to typically occur with aging. At the final TOF-MRA measurement the animals were with 17 months relatively old given the average live span of rats.

The defects observed by TOF-MRA indicate the loss or the reduction of vascular blood flow in the respective brain region most likely caused by pathological alterations of vessel morphology. Damages of target organs caused by antibodies directed at G protein-coupled receptors were reported in numerous animal studies [5], [7], [8], [13], [16]. Rats immunized with peptides corresponding to the second extracellular loop of the α_1_-AR showed signs of cardiac remodeling, developed left ventricular damages with diastolic dysfunction and vascular damages in aorta and mesenteric artery [7]–[9]. Autoantibodies to the α_1_-AR isolated from hypertensive patients and α_1_-AR antibodies raised in rabbits induced signaling pathways involved in cardiac and vascular remodeling [9].

The importance of antibodies directed at the α_1_-AR in vascular pathology was shown for peripheral vessels such as aorta and mesenteric artery [8], [9], [13]. Here we demonstrate the deleterious action of α_1_-AR antibodies in the vasculature of the central nervous system of rats. Agonistic antibodies to the α_1_-AR were found to be associated with different forms of hypertension and type 2 diabetes, widespread diseases with a crucial role of vascular impairments [9]–[12]. Vascular damages are most likely the mechanism linking type 2 diabetes to cerebrovascular diseases [18], [19]. The present data extend the importance of antibodies to the α_1_-AR to diseases which directly affect the central nervous system such as stroke and dementia. They may be part of the mechanism of age-related cerebral small vessel diseases (CSVD) affecting blood brain barrier (BBB) integrity and known to be a major cause for stroke-like symptoms and pre-stages of vascular dementia [20]. Progressing vascular damages worsen blood and oxygen supply in brain regions and eventually cause stroke. Notably, there is a link between stroke and dementia. Patients who suffered from stroke have an increased risk of faster and earlier developing dementia [21]. Dementia of the vascular and the Alzheimer’s type are difficult to discriminate, and typically they develop coincidentally. Recently we identified autoantibodies predominantly directed at the α_1_-AR in the circulation of patients with Alzheimer’s/vascular dementia [22]. The mechanisms underlying Alzheimer’s dementia are still a matter of dispute. There is growing evidence that also Alzheimer’s disease is primary a vascular disease [1]. Damages in brain vessels may attenuate the clearance of β-amyloid from neuronal tissue in patients, a mechanism which increasingly becomes acknowledged to be crucial for the progression of the disease [23].

Taken together, in the present study we provide evidence for the potential of α_1_-AR antibodies to cause damages of brain vessels in a rat model. Our data suggest the pathogenic significance of autoimmunity to the α_1_-AR for diseases of the central nervous system such as stroke and dementia. They further affirm previous evidences that α_1_-AR autoantibodies are important players in vascular pathology.
